# Exploring the Well-being of Health Care Workers During the COVID-19 Pandemic: Protocol for a Prospective Longitudinal Study

**DOI:** 10.2196/32663

**Published:** 2021-09-27

**Authors:** Jenny J W Liu, Anthony Nazarov, Rachel A Plouffe, Callista A Forchuk, Erisa Deda, Dominic Gargala, Tri Le, Jesse Bourret-Gheysen, Vanessa Soares, Maede S Nouri, Fardous Hosseiny, Patrick Smith, Maya Roth, Arlene G MacDougall, Michelle Marlborough, Rakesh Jetly, Alexandra Heber, Joy Albuquerque, Ruth Lanius, Ken Balderson, Gabrielle Dupuis, Viraj Mehta, J Don Richardson

**Affiliations:** 1 MacDonald Franklin Operational Stress Injury Research Centre Lawson Health Research Institute St. Joseph's Health Care London London, ON Canada; 2 Department of Psychiatry Schulich School of Medicine and Dentistry Western University London, ON Canada; 3 Centre of Excellence on Post-Traumatic Stress Disorder and Related Mental Health Conditions Royal Ottawa Mental Health Centre Ottawa, ON Canada; 4 St. Joseph’s Operational Stress Injury Clinic Greater Toronto Area, ON Canada; 5 Canadian Armed Forces Ottawa, ON Canada; 6 Veterans Affairs Canada Ottawa, ON Canada; 7 Department of Psychiatry Faculty of Medicine University of Toronto Toronto, ON Canada

**Keywords:** COVID-19, health care worker, pandemic, mental health, wellbeing, survey, design, longitudinal, prospective, protocol, challenge, impact, distress, perception

## Abstract

**Background:**

Health care workers (HCWs) have experienced several stressors associated with the COVID-19 pandemic. Structural stressors, including extended work hours, redeployment, and changes in organizational mandates, often intersect with interpersonal and personal stressors, such as caring for those with COVID-19 infections; worrying about infection of self, family, and loved ones; working despite shortages of personal protective equipment; and encountering various difficult moral-ethical dilemmas.

**Objective:**

The paper describes the protocol for a longitudinal study seeking to capture the unique experiences, challenges, and changes faced by HCWs during the COVID-19 pandemic. The study seeks to explore the impact of COVID-19 on the mental well-being of HCWs with a particular focus on moral distress, perceptions of and satisfaction with delivery of care, and how changes in work structure are tolerated among HCWs providing clinical services.

**Methods:**

A prospective longitudinal design is employed to assess HCWs’ experiences across domains of mental health (depression, anxiety, posttraumatic stress, and well-being), moral distress and moral reasoning, work-related changes and telehealth, organizational responses to COVID-19 concerns, and experiences with COVID-19 infections to self and to others. We recruited HCWs from across Canada through convenience snowball sampling to participate in either a short-form or long-form web-based survey at baseline. Respondents to the baseline survey are invited to complete a follow-up survey every 3 months, for a total of 18 months.

**Results:**

A total of 1926 participants completed baseline surveys between June 26 and December 31, 2020, and 1859 participants provided their emails to contact them to participate in follow-up surveys. As of July 2021, data collection is ongoing, with participants nearing the 6- or 9-month follow-up periods depending on their initial time of self-enrollment.

**Conclusions:**

This protocol describes a study that will provide unique insights into the immediate and longitudinal impact of the COVID-19 pandemic on the dimensions of mental health, moral distress, health care delivery, and workplace environment of HCWs. The feasibility and acceptability of implementing a short-form and long-form survey on participant engagement and data retention will also be discussed.

**International Registered Report Identifier (IRRID):**

DERR1-10.2196/32663

## Introduction

### Background

Throughout the COVID-19 pandemic, health care workers (HCWs) have served on the frontlines of disease management and response. In their roles, HCWs have experienced increased workloads, risks of redeployment, and exposure to SARS-CoV-2 while caring for the surge of patients. At the same time, HCWs may be concerned about the safety and well-being of themselves, their families, and their loved ones. Within a larger context, HCWs are situated in working environments that may be experiencing rapid changes, such as implementation of new safety protocols, adapting to telehealth service delivery, or contending with redeployment. These changes in the workplace are further compounded by increasingly challenging work environments, where HCWs may encounter difficult moral-ethical dilemmas (eg, tending to patients without adequate personal protective equipment (PPE), providing services on platforms unfamiliar to the provider and patients), which may have severe and enduring consequences for their mental health and well-being.

Research following the 2003 severe acute respiratory syndrome (SARS) epidemic illustrates the significant and persevering distress that HCWs may experience in the aftermath of an infectious outbreak. Among SARS survivors, HCWs experienced elevated symptoms of anxiety one month following SARS recovery [1] and higher levels of stress, depression, and anxiety at one-year postoutbreak [2] compared to non-HCW survivors. Evidence from the SARS epidemic also highlighted the vulnerability of those working on the frontlines, including job stress related to managing changes to working environments, feelings of loneliness and social isolation, and anxiety and fear in response to increased exposures to the virus [3,4]. In comparison to hospital administrative staff, frontline HCWs reported significantly higher psychological impairment, insomnia, and exhaustion [5].

Studies conducted early during the COVID-19 pandemic similarly found that HCWs on the front line were more severely distressed compared to nonfrontline HCWs [6-8]. In other cross-sectional studies, evidence also points to the devastating toll of the pandemic on the mental health of HCWs. In a study of nurses and physicians in Wuhan, China, over 60% of respondents reported concerning mental health symptoms across standardized measures of anxiety, depression, and sleep [9]. Similar increases in psychological distress, burnout, and worsened mental health were reported in frontline HCWs in other countries [10-13].

Some of the distress experienced by HCWs could be ethical or moral in nature. HCWs’ experiences during the pandemic involve making difficult decisions that may not always be aligned with their ethical or moral values. These may involve tending to patients without appropriate PPE, balancing increases in patient caseloads and potentially compromising the quality of care provided, having to make difficult decisions to turn away patients without care due to shortages of hospital beds or ventilators, and disagreements or conflicts arising from the allocation of lifesaving treatments or vaccines [14,15]. Expanding beyond organizational levels, rapidly changing public health policies, and perceived delays in responses from leaders, employers, and municipal, provincial, and federal governments may perpetuate feelings of distrust and betrayal, further triggering complex emotional reactions. Indeed, moral distress may arise when individuals find themselves in difficult emotional states when the perceived ethical actions deviate from what they may be tasked to do. The frequency and impact of morally distressing events may be amplified during the current pandemic [14,15]. If unaddressed, these instances of moral distress can lead to moral injury, defined as the psychological distress resulting from transgressing one’s moral beliefs or standards through action or inaction [16,17]. Despite evidence of mental distress, little empirical attention has been paid to the examination of moral-ethical dilemmas and associated moral distress during current and past epidemics and pandemics.

Further, the myriad of challenges HCWs face at the individual and organizational levels greatly threaten their physical and mental health, as well as their professional development. Specifically, increased rates of absenteeism reported by HCWs not only contribute to added burden of care for colleagues but more importantly highlight the systemic need for additional support for HCWs [18]. Meanwhile, reviews suggest that increased workloads, occupational stress and burnout, and organizational changes are expected to pose critical challenges in future to the long-term retention of HCWs [19]. Taken together, research is urgently needed to understand HCWs’ experiences during the COVID-19 pandemic, including challenges and changes in the workplace, moral-ethical dilemmas, evolving occupational duties, standards of care, service delivery, and the effects of COVID-19 on dimensions of mental health and well-being.

### Research Aims

This paper describes the protocol and initial response rates for a longitudinal study seeking to capture the unique experiences, challenges, and changes faced by HCWs during the COVID-19 pandemic. The study was launched in June 2020 and is ongoing. Future publications from this study will use the data collected to explore (1) the impact of COVID-19 on the mental well-being of HCWs, (2) perceptions of and satisfaction with delivery of care, and (3) how changes in work structure are tolerated among HCWs providing services.

## Methods

### Summary of Design

Our study employs an observational, prospective, longitudinal panel design using the web-based data collection platform Research Electronic Data Capture (REDCap). Participants completed questionnaires at baseline and will be completing follow-up questionnaires at 3-month intervals for a total of 18 months. Interested participants self-selected into an open survey and chose to complete a short version or long version of the survey at baseline. The protocols of this study were reviewed and approved by the research ethics board at Western University (WREM 115894) and Lawson Health Research Institute (REDA 9968). Details of the protocol are reported below following the general guidelines from the Checklist for Reporting of Results of Internet E-Surveys (CHERRIES) [20].

### Participant Selection and Recruitment

A convenience snowball sampling approach is used to recruit HCWs. Recruitment methods include word of mouth, emails to professional networks, web-based advertisement through the Lawson Health Research Institute, social media, participant recruitment websites (eg, ParticipAid [21]), and targeted media releases. The representativeness of participants was monitored throughout the recruitment period. Recruitment efforts are adjusted to target specific regions or segments to improve the representation on dimensions of gender, region, and occupational distribution of health care workers within Canada. Participants include male and female English- and French-speaking HCWs with a minimum age of 18 years. HCWs are defined as individuals who provide health care treatment and advice based on formal training and experience or who work to directly support those providers in a clinical setting. Participating HCWs must be currently working in Canada or have worked in Canada as an HCW at some point in time between the start of the COVID-19 pandemic (March 2020) and the start of data collection (June 26, 2020). Participation in the study is voluntary, and participants are not compensated for survey completions.

### Procedure

The study duration is catered to participant availability and varies depending on whether the participant selected the short-form or long-form survey (Table 1). Both English and French versions of the survey are available based on the language preference of participants. Validated scales in French are used where available, and in the absence of translated and validated versions, translation is completed by professional translators with certificates of translation provided. Informed consent is obtained through a Letter of Information (LOI), presented to participants at the beginning of the survey at baseline, and again during each of the follow-up surveys. The LOI is presented on REDCap, and participants are informed that their consent is implied should they proceed to the following pages of the survey.

A short-form version of the survey is available at baseline (approximately 10 minutes) and consists of 6 measures. A longer option, consisting of 12 measures, is available for participants who indicate they have the time (approximately 15-25 minutes). The long version can be completed immediately at baseline or returned within a 6-week window after beginning the baseline measures. Following baseline, participants are requested to complete a follow-up survey (approximately 15 minutes) every 3 months for a period of 18 months (see Table 1). To save time, participants can skip certain modules if there was no change from previous time points (eg, if their employment status did not change). Participants can also review and change answers before advancing to the following page. 

**Table 1:** Data collection tools for the short-form, long-form, and follow-up surveys.

Collection of assessments/domains			Short form		Long form		Follow-up
Demographics			✓		✓		
Work-related changes/appraisals			✓		✓		✓
Telehealth experiences					✓		✓
Organizational response			✓		✓		✓
COVID-19 exposure/concerns			✓		✓		✓
**Mental health**							
	Patient Health Questionnaire-9			✓		✓	
	Generalized Anxiety Disorder-7 scale			✓		✓	
	Posttraumatic Stress Disorder Checklist for DSM-5^a^	✓		✓		✓	
	Well-Being Index			✓		✓	
**Moral injury, ethical climate, and moral reasoning**							
	Measure of Moral Distress for Healthcare Professionals	✓		✓		✓	
	Moral Injury Outcome Scale			✓			
	Ethical Environment Questionnaire			✓		✓	
	Oxford Utilitarianism Scale			✓			

^a^DSM-5: *Diagnostic and Statistical Manual of Mental Disorders*, 5th edition.

### Measures

The web-based survey was constructed in consultation with international research teams and collaborators to evaluate the crosscultural effects of the COVID-19 pandemic. Measures were selected to ensure comparability across countries. In addition, a literature search was conducted to identify potential novel measures related to the assessment of effects of the COVID-19 pandemic. The usability and functionality of the survey were tested by the research team, including adaptive questioning, branching functions, and longitudinal response collections. Questions were presented in the same order, as shown in Table 2.

**Table 2:** Categories and types of questions included in the survey.

Category	Question types
Basic demographics	All participating HCWs^a^ are asked to report their province or territory of residence, age, and ethnicity.^b,c^ Participants choosing to complete the long form questionnaire are also asked to report their gender, marital status, characteristics of area of residence (eg, rural or city), and education level.
Work-related/telehealth	Participants are asked about work demographics, the proportion of their time spent working on-site versus remotely, the proportion of their time working directly with patients, and whether they provided care to patients with suspected or confirmed COVID-19. HCWs providing services remotely are asked closed- and open-ended questions about the changes to their delivery of care and their experiences with telehealth. The telehealth questions were derived from a University of Missouri quality improvement survey and other research exploring the application of telehealth and telemedicine in various populations [22-25].
Organizational response to COVID-19	Participants are asked to self-report on the effectiveness and satisfaction with the support and communication of their organization in response to the COVID-19 pandemic. Items were drawn from the Pandemic Experiences and Perceptions Survey [26], a measure created in response to the COVID-19 pandemic. This survey measures organizational response to the pandemic in the domains of disruption, resource adequacy, COVID-19 risk perception, positive work life impact, and leadership [26]. For the purpose of our study, we are collecting data on the resource adequacy, risk perception, positive work life impact, and leadership domains.
COVID-19 exposure/concerns	Participants are asked to report their history of suspected or confirmed exposure to and infection with COVID-19 individually and for family members, as well as any associated direct impact the infections had on them. The items were adapted from the Coronavirus Health Impact Survey, which was developed based on ongoing research and collaborations between the National Institute of Mental Health Intramural Research Program Mood Spectrum Collaboration, the Child Mind Institute, the New York State Nathan S Kline Institute for Psychiatric Research, and researchers from Johns Hopkins University [27].
Mental health questionnaires	Symptoms of mental distress are evaluated using self-report measures, including a measure of depression (the Patient Health Questionnaire-9 [28]),^b^ posttraumatic stress disorder (the PTSD Checklist for DSM-5 [29]), generalized anxiety (the Generalized Anxiety Disorder-7 scale [30]),^b^ and workplace well-being and burnout (Well-Being Index [31]).^b^ Additional questions were included to determine the extent to which mental health symptoms may have been influenced or exacerbated by the pandemic.
Moral distress, ethical climate, and moral reasoning	Moral distress, moral injury, ethical workplace climate, and moral reasoning are assessed using self-report measures. Perceptions of the general ethical climate in the HCWs’ workplaces will be evaluated using the Ethics Environment Questionnaire [32]. Individual experiences with specific morally distressing situations (eg, “Watch patient care suffer because of a lack of provider continuity”) are evaluated using the Measure of Moral Distress for Healthcare Professionals [33]. Multidimensional moral injury is evaluated using the Moral Injury Outcome Scale^b^ [34]. Moral-ethical decision-making tendencies are assessed using the Oxford Utilitarianism Scale^b^ [35].

^a^HCW: health care worker.

^b^Represents a measure that is available only on the long-form version of the web-based survey.

^c^Ethnicity was added at a later time via an ethics amendment to the original protocol, and a portion of participants who are completing the 12-month follow-up survey will have the option to answer questions on their racial and ethnic background.

### Data Analytic Plan

#### Descriptive and Exploratory Analyses

Mixed methods descriptive and exploratory analyses will be conducted to understand the state of mental well-being and moral distress of sampled HCWs. Quantitative descriptive statistics will examine age, gender, education, occupation, illness-related variables (eg, whether currently or formerly positive for COVID-19), psychological and moral variables, and satisfaction with telehealth. These will include general descriptive statistics, measures of internal consistency, correlational analyses, and group-based analyses on similarities and differences. Exploratory analyses will include hierarchical multivariate analyses, structural equation modelling, and cluster analyses to determine manifestations of mental and/or moral distress. Qualitative, open-ended data, including participant descriptions of changes to their care delivery, impact of moral distress, and general feedback on organizational support and pandemic responses will be analyzed using content and thematic analysis.

#### Longitudinal Analyses

Data at baseline and each of the follow-up periods will be analyzed using exploratory and confirmatory mixed effects modeling and latent growth modeling. Longitudinal analyses will explore changes in dimensions of mental health, moral injury, and distress in relation to care delivery and/or work settings, organizational responses, and COVID-19-related changes and exposures over time.

## Results

### Survey Completion and Representativeness

**Figure 1:**
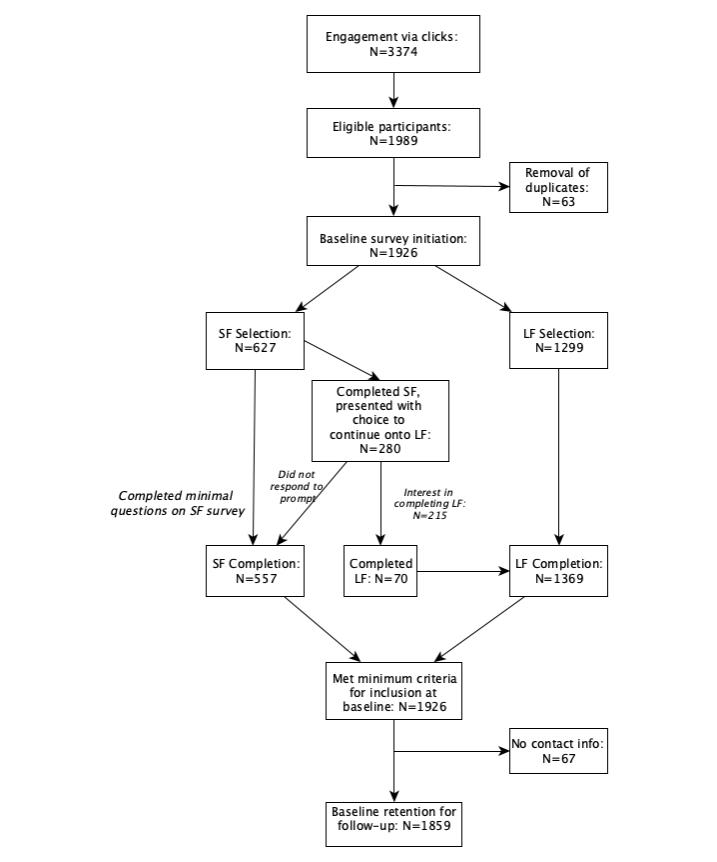
Participant flow for baseline completion rates. Info: information; LF: long-form survey; SF: short-form survey.

A total of 1926 participants completed baseline surveys between June 26 and December 31, 2020. Of these, 1859 participants provided their emails for follow-up survey invitations. The majority (71%, n=1299) of baseline participants initially selected the long-form survey. Subsequent prompts encouraged participants completing the short-form surveys to complete the long version, which resulted in 25% conversion of those prompted and an overall increase in long-form survey data. Figure 1 details the flow of survey completion from initial interest and engagement via survey links.

We further examined the preliminary representativeness of our baseline sample against a Canadian national database of health care workforce metadata with over 37 million health care workers on the distribution of top professions and genders [36]. Relative to the national sample, which comprises roughly 68% nurses and 14% physicians, our baseline sample from self-reported professions included 40.27% (n=557) nurses and 3.90% (n=54) physicians, representing differences of 28% and 10%, respectively. For other top health care disciplines, such as personal support workers, paramedics, physical therapists, and social workers, our sample was relatively representative, with differences in percentage distributions of 1.2% to 5.6%. Finally, female nurses were underrepresented in our sample by a percentage difference of 32%, whereas female HCWs in other disciplines and male HCWs were relatively representative in relation to the national sample (percentage differences of <1% to 7%).

### Participant Data Retention

**Figure 2:**
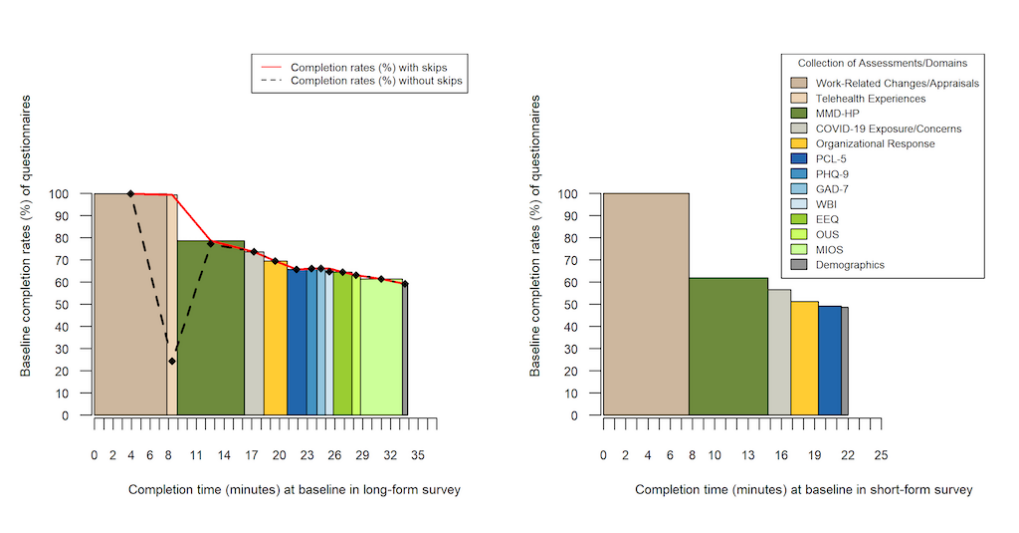
Completion rates of individual questionnaires at baseline.

Depending on whether participants completed the short-form or long-form survey at baseline, completion time varied by approximately 12 minutes, with the long-form survey taking a median of 34 minutes and the short-form survey taking a median of 22 minutes to complete. Survey completion also varied by questionnaire. For the long-form survey, baseline completion for initial eligibility, work-related, and telehealth items were completed by nearly all participants (100%), with participation declining to <80% for COVID-19–related questions and to <70% for mental health– and moral distress–related self-report questionnaires (n/N values are not reported for the percentages here, as they represent changes in rates of completion as opposed to changes in sample size). This trend was similar for short-form questionnaire completion, but with a steeper decline, and with completion of the full set of questionnaires between 50% and 60% (see Figure 2). Although the dropout rate was comparable to those found in previous research evaluating completion rates of web-based surveys in relation to the length of the surveys, this study was able to maintain 100% initial completion for the first section of questions about work-related changes and responses to COVID-19, whereas others have reported 10% instantaneous dropout [37]. Furthermore, although previous studies examined participation under regular circumstances using university student samples, this study sampled HCWs who are likely time-restricted because of their busy schedules. Taken together, the choice between short- and long-form surveys and the option to convert to the long form is a promising approach to maximize participation and retention.

Finally, based on the date of self-selection and enrollment in the study, participants receive system-generated links to complete follow-up surveys every three months. The baseline retention with email addresses (N=1859) is being used as a reference point to evaluate subsequent participant retention, attrition, and sensitivity analyses during each of the follow-up periods. As of July 2021, a total of 848/1859 participants (45.6%) had completed the 3-month follow-up, whereas 1011/1859 (54.4%) missed the response window. Data collection is ongoing into the 6-month and 9-month follow-up periods. Full data collection for the longitudinal study is expected to be completed by August 8, 2022. 

## Discussion

This paper details the protocol for a longitudinal study that will examine the impact of the COVID-19 pandemic on the mental well-being of HCWs, with a focus on moral distress, perceptions of and satisfaction with delivery of care, and perceptions of changes in work structure among HCWs providing services. Using convenience snowball sampling and diverse recruitment platforms, this study is reaching a large national sample of HCWs from a range of disciplines and backgrounds. Participants recruited are relatively diverse and comparable to national samples of HCW distributions. Following baseline completion, we retained 1859 HCWs for subsequent longitudinal follow-ups. The longitudinal data will provide important profiles of HCWs during key milestones of the pandemic in Canada and offer insights in understanding and predicting the development or worsening of mental health and moral injury over time as the pandemic persists. In particular, the collected data will shed light on the organizational and environmental stressors and their associations with changes in the experiences of HCWs with moral dilemmas, moral distress, and mental well-being.

A strength of this study that may contribute to the large sample size for both recruitment and retention is the option to complete either the short or long form of the survey. With the choice to select either form, we sought to reduce barriers of participation as a result of survey fatigue and self-perceived time restrictions to accommodate the busy schedules of HCWs. To further encourage completion of the long-form survey, we also implemented prompts to encourage those who completed the short-form survey to complete the long-form survey in subsequent follow-ups. This option yielded a 25% success rate in conversion to the long-form survey. This represents a relatively novel approach, which was designed to maximize survey retention while minimizing barriers [38]. A challenge in this process was the time difference in completion between short- and long-form surveys. Based on data completion rates and times at baseline, the difference between the two surveys is estimated to range between 5 and 15 minutes, with the short-form survey taking a median of 22 minutes to complete. Given the initial time commitment required for the short-form survey, this may have discouraged some of the participants from converting to the long-form survey based on their perceived time restraints.

As of July 2021, data collection is ongoing, with participants nearing the 6- or 9-month follow-up periods depending on their initial time of self-enrollment. This study demonstrates the utility and feasibility of offering both a short-form and long-form survey for the collection of prospective, longitudinal data from HCWs. This format of recruitment and data collection may be useful when implemented with other populations experiencing time restrictions or busy schedules. Finally, this study will offer key insights into the mental well-being and moral challenges of HCWs as they cope with the ongoing pandemic. The findings will lend a voice to the HCWs and their unique experiences of challenges and change during the protracted pandemic and associated restrictions. Knowledge gained will better equip us to anticipate and prepare for future challenges, such as long-term support and retention of HCWs. Armed with this knowledge, policy makers and clinicians can make evidence-based decisions to prevent and mitigate the risks to the psychological well-being of HCWs both generally and specifically as they recover from the pandemic.

## Abbreviations

CoE: Centre of Excellence on Post-Traumatic Stress Disorder and Related Mental Health Conditions
HCW: health care worker
LOI: Letter of Information
PPE: personal protective equipment
REDCap: Research Electronic Data Capture
SARS: severe acute respiratory syndrome
